# Functional Diversification and Specialization of Cytosolic 70-kDa Heat Shock Proteins

**DOI:** 10.1038/srep09363

**Published:** 2015-03-20

**Authors:** Chelsea McCallister, Matthew C. Siracusa, Farzaneh Shirazi, Dimitra Chalkia, Nikolas Nikolaidis

**Affiliations:** 1 Department of Biological Science, Center for Applied Biotechnology Studies, and Center for Computational and Applied Mathematics, California State University, Fullerton, Fullerton, CA 92834; 2 Center for Mitochondrial and Epigenomic Medicine, The Children's Hospital of Philadelphia Research Institute, Philadelphia, PA

## Abstract

A fundamental question in molecular evolution is how protein functional differentiation alters the ability of cells and organisms to cope with stress and survive. To answer this question we used two paralogous Hsp70s from mouse and explored whether these highly similar cytosolic molecular chaperones, which apart their temporal expression have been considered functionally interchangeable, are differentiated with respect to their lipid-binding function. We demonstrate that the two proteins bind to diverse lipids with different affinities and therefore are functionally specialized. The observed lipid-binding patterns may be related with the ability of both Hsp70s to induce cell death by binding to a particular plasma-membrane lipid, and the potential of only one of them to promote cell survival by binding to a specific lysosomal-membrane lipid. These observations reveal that two seemingly identical proteins differentially modulate cellular adaptation and survival by having acquired specialized functions via sequence divergence. Therefore, this study provides an evolutionary paradigm, where promiscuity, specificity, sub- and neo-functionalization orchestrate one of the most conserved systems in nature, the cellular stress-response.

A central question in molecular evolution is how sequence divergence of duplicated genes, either by mutations or domain shuffling, results in functional changes that enable organisms to adapt to their environment and thrive. At the cellular level the same essential question relates to the ability of cells and cellular systems to adapt and survive homeostatic imbalances due to stress.

To maintain homeostasis the cellular stress response (CSR) has evolved^1^. The CSR is composed by several protein networks, which by interacting with each other and their ligands, e.g., proteins, lipids, or nucleic acids, regulate cell survival^1^,^2^. Molecular chaperones are key regulators of the stress response system and alterations in their function have direct physiological consequences. The primary functions of several such proteins have been retained for several millions or billions of years^1^,^2^,^3^.

The 70-kD heat shock proteins (Hsp70s) are one of the most compelling examples of proteins functioning in the CSR in which the primary function, protein folding and refolding, has been retained from bacteria to humans^3^,^4^,^5^. Yet, in most eukaryotes certain Hsp70s acquired specialized functions that allowed cells and organisms to adapt, survive, and thrive in various environments. For example, several *hsp70s* are expressed constitutively in all cell types, others exhibit a tissue-specific constitutive expression pattern, and yet some others are expressed only in response to stress, e.g., heat-shock^3^,^4^,^5^,^6^. Furthermore, different members of the family are localized and function in different subcellular compartments, e.g., cytosol, endoplasmic reticulum, mitochondria, or the plant chloroplasts (Fig. 1)^3^,^4^,^5^,^6^.

Unlike other heat-shock proteins, such as the Hsp40s, which evolved specialized functions via sequence divergence and domain acquisition^7^, Hsp70s have diverged exclusively via nucleotide mutations^6^,^8^,^9^. Mutations at the coding regions of the genes resulted in specific amino acid changes that allowed the proteins to be targeted and retained to particular organelles^3^,^5^,^6^,^8^,^9^, while mutations at the *hsp70s'* promoter regions resulted in different expression patterns, e.g., constitutive or induced by stress^3^,^5^,^6^,^8^,^9^. The coding region mutations seem to have occurred once or a few times during eukaryotic evolution, as the differently localized Hsp70s form deep phylogenetic clades dating back to the very first eukaryotes (Fig. 1)^3^,^4^,^6^,^8^,^9^. In contrast, the promoters' sequence changes, especially in the cytosolic *hsp70s*, have occurred multiple times during eukaryotic evolution, as it appears that heat-inducibilty has evolved independently more than once in different phylogenetic lineages (Fig. 1)^8^,^10^.

Despite their temporal and spatial differentiation, all Hsp70s seem to perform the same chaperone functions. Therefore, it has been postulated that Hsp70s do not possess any functional specialization with respect to their protein clients^5^,^11^, with some notable exceptions, e.g., the cytosolic SSBs and the mitochondrial SSC2 in yeast, which function exclusively in nascent polypeptide folding^12^ and iron homeostasis^13^, respectively.

Yet, several reports have shown that specific human and mice Hsp70s localize at the plasma membrane (PM), associate with lipid-rafts, and bind to specific lipids^14^,^15^,^16^,^17^,^18^,^19^,^20^,^21^,^22^,^23^,^24^,^25^,^26^,^27^,^28^. Although the mechanism of these Hsp70s' functions is less well characterized and understood compared to their protein chaperone functions, it has been demonstrated that the interaction of Hsp70s with membranes and lipids has direct physiological outcomes. These include activation of the immune system, viral entry, stabilization of the lysosomal membrane, microautophagy, trafficking of anandamide from the plasma membrane to internal cellular compartments, and promotion of cell apoptosis^14^,^15^,^16^,^17^,^18^,^19^,^20^,^21^,^22^,^23^,^24^,^25^,^26^,^27^,^28^,^29^. Still, it remains unknown how the lipid-binding function evolved, whether it is promiscuous or not, and if it functionally differentiates Hsp70s.

To answer some of these questions and shed light on the evolution of the Hsp70 lipid-binding functions we used two mammalian cytosolic proteins, HspA1A and HspA8, as a model system. These proteins combine five desirable characteristics that make them a suitable model to study the evolution of the Hsp70-lipid function. First, the genes coding for HspA1A and HspA8 are heat inducible and constitutively expressed, respectively (Fig. 1). Second, *hspA1A* and *hspA8* are evolving differently, the former following a concerted and the latter a birth-and-death model of evolution (Fig. 1). Third, these proteins are considered functionally interchangeable, because they bind to their client proteins without showing any kind of specificity and have similar subcellular localization^5^,^6^,^7^. Fourth, both proteins are found at the PM, are associated with lipid-rafts, and are secreted at the extracellular space. And fifth, both chaperones bind to the PM lipid phosphatidylserine (PtdSer) *in vitro* and *in vivo*^14^,^16^,^17^,^18^,^19^,^30^.

Furthermore, there is evidence that lipid binding may differentiate HspA1A and HspA8. For example, it has been shown that PtdSer-binding differentiates HspA1A and HspA8, because, although both proteins induce the aggregation of liposomes containing PtdSer, the reactions have different rates^30^. Additionally, it was shown that HspA1A binds to bis(monoacylglycerol)phosphate (BMP) and stabilizes the lysosomal membrane^29^, whereas HspA8 binds to PtdSer and mediates microautophagy^31^. The former report, in particular, demonstrated that although both HspA1A and HspA8 are entering the cells via endocytosis, only HspA1A rescues the lysosomal membrane, while HspA8 does not. The above reports suggest that although both proteins bind to PtdSer and regulate cell survival, only HspA1A binds to BMP while HspA8 does not.

Based on the above observations we hypothesized that lipid-binding is a conserved function of Hsp70s and in contrast to their promiscuous chaperone functions, HspA1A and HspA8 are differentiated with respect to their lipid-binding properties. To test this hypothesis and provide quantitative evidence of the Hsp70-lipid interaction we generated recombinant HspA1A and HspA8 proteins and determined their lipid binding properties using both qualitative and quantitative assays.

## Results

### Primary and Tertiary Sequence Analysis

Mouse HspA1A and HspA8 are 84% identical at the protein level and have almost identical folding patterns and three-dimensional (3D) structures (Fig. 2 and Supplementary Fig. S1). Despite the high level of structural conservation especially at the nucleotide-binding domain of HspA1A and HspA8, their surface electrostatic potential is quite different. The presence of differentially positively charged regions at the proteins' surface suggests that their interaction with negatively charged lipid heads should be dissimilar.

### Generation, Expression, and Purification of Functional HspA1A and HspA8 Gene Products

We employed the polymerase chain reaction to amplify the full length HspA1A and HspA8 gene sequences using specifically designed oligonucleotide primers. We then cloned, expressed, and purified the amplified products (Fig. 3a, b). The apparent molecular weights of each protein were determined by SDS-PAGE. Transfer of the purified protein products to nitrocellulose and probing with anti-His antibody demonstrated equal reactivity with all products, showing a single major immunoreactive species in each case (Fig. 3b).

We then tested whether the recombinant proteins were functional by testing three typical Hsp70 functions. Specifically, we determined that the recombinant proteins prevent protein aggregation, promote protein refolding, and hydrolyze ATP (Fig. 3c–e).

### Lipid-binding Screening

To determine the spectrum of HspA1A potential lipid ligands we screened arrays of 36 lipids using the protein-lipid overlay (PLO) assay as implemented by Eschelon Biosciences. This initial screen showed that recombinant HspA1A binds to several anionic phospholipids, phoshpatidylserine (PtdSer), phoshpatidic acid (PtdOH), phoshpatidylglycerol (PtdGro), cardiolipin (Ptd2Gro), and several phoshpatidylinositols (PtdIns), and a single sphingolipid sulfogalactosylceramide (sulfatide; GalCer-I^3^-sulfate) (Fig. 4a). To validate these initial results, get a semi-quantitative view of the lipid binding, and test whether both proteins bind to the same lipids we used an alternative form of the PLO assay by spotting different lipids and lipid amounts on nitrocellulose membranes. These assays confirmed the results of the first screen and strongly suggested that both HspA1A and HspA8 bind specifically to several anionic lipids (Fig. 4b).

To further confirm the above results and gain quantitative information of the binding of both proteins to specific lipids we used the lipid vesicle sedimentation (LVS) assay. For this assay we first determined that the protein does not precipitate (no lipid controls) and also used several lipids that did not show binding to either protein as negative controls. In all experiments phoshpatidylcholine (PtdCho), a lipid for which both proteins show minimal binding, was used as the basal lipid to generate small unilamellar vesicles (liposomes) with the lipid of interest (Fig. 5 and Supplementary Fig. S2). This assay validated the original results and revealed that in several cases the amount of lipid binding is significantly different between HspA1A and HspA8, e.g., PtdSer, BMP, GalCer-I^3^-sulfate, Ptd2Gro, Phosphatidylinositol 3,5-bisphosphate [PtdIns(3,5)P_2_], and Phosphatidylinositol (3,4,5)-triphosphate [PtdIns(3,4,5)P_3_] (Fig. 5).

### Lipid-binding Kinetics

To mechanistically characterize the lipid-binding properties of HspA1A and HspA8 we generated binding curves and determined binding kinetics (Fig. 6, Table 1, and Supplementary Tables S1 and S2). The results of these assays revealed that when the two proteins bind to the same lipid they have different: (a) affinities, values of apparent dissociation constants (Kd), (b) maximal binding (Bmax), and (c) in some cases, theoretical binding models. Furthermore, the significantly different dissociation values imply different lipid specificities, and the distinctive theoretical models suggest mechanistically altered lipid binding.

In particular our data show that both HspA1A and HspA8 bind to PtdSer with relatively high affinity (low K_d_ values; K_d_ = 14 μM and 72 μM, respectively). However, both the affinity and maximal binding of HspA1A to PtdSer are significantly different compared to HspA8 (Fig. 6 and Table 1). Also, the theoretical models that best describe the interaction between the proteins and PtdSer suggest the presence of two binding sites on HspA1A and one site on HspA8 (Table 1 and Supplementary Table S2).

The affinity of HspA1A for BMP (K_d_ = 148 μM) is more than four times higher than that of HspA8 (K_d_ = 598 μM) for the same lipid (Fig. 6 and Table 1). The model that best describes the binding of both proteins to BMP is a modified Hill model (cooperative binding). The Hill coefficient, a, is smaller than 1 in the case of HspA1A, suggesting that the binding of one BMP molecule to the protein decreases its affinity for other BMP molecules (negatively cooperative binding). In contrast, the Hill coefficient is higher than 1 in the case of HspA8 suggesting positively cooperative binding (the binding of one BMP molecule to HspA8 increases its affinity for other BMP molecules) (Table 1).

HspA1A's affinity for PtdOH is 151 μM, which is double the affinity of HspA8 for the same lipid (K_d_ = 341 μM). The binding of both proteins to PtdOH is best described using the Hill cooperative model (Supplementary Tables S1 and S2). Based on this model, HspA1A shows positively cooperative binding (a = 2.1) and HspA8 negatively cooperative binding (a = 0.6).

The affinity of HspA8 for PtdGro is five times higher (K_d_ = 40 μM) than that of HspA1A (K_d_ = 203 μM). In this case, the binding of both proteins to PtdGro is best described by the single site saturation model, suggesting the presence of one binding site.

In the case of GalCer-I^3^-sulfate, HspA1A and HspA8 show different affinities and maximal binding, with HspA8's values (K_d_ = 85 μM; B_max_ = 73.6) being significantly higher than HspA1A's (K_d_ = 111 μM; B_max_ = 56.7). Also, the theoretical models that best describe the interaction between the proteins and GalCer-I^3^-sulfate suggest the presence of cooperative binding in the case of HspA1A and a non-cooperative binding (one site saturation) in the case of HspA8 (Supplementary Tables S1 and S2).

Lastly, both proteins bind to Ptd2Gro, a lipid found exclusively in the eukaryotic mitochondrial membrane and the bacterial PM, with comparable affinities and mechanism (Supplementary Tables S1 and S2).

## Discussion

Three major observations summarize our results on the lipid-binding properties of the mouse HspA1A and HspA8. First, both proteins bind to several anionic lipids (Figs. 4 and 5). Second, the affinities of the interactions are relatively low (Table 1 and Table 1 and Supplementary Table S1). And third, both proteins bind to lipids with different affinities and specificities (Fig. 6, Table 1 and Supplementary Tables S1 and S2).

The binding to anionic lipids could be the result of non-specific purely electrostatic interactions. However, this explanation seems highly unlikely because the amount of protein bound to a particular lipid does not increase as the charge increases. For example, the binding of HspA1A to Phosphatidylinositol 4-phosphate [PtdIns(4)P], which has a charge of −2, is much higher than to PtdIns(3,4,5)P_3_, which has a charge of −5. In addition to our data, other reports have shown that the interaction of HspA1A with particular lipids, e.g., PtdSer and PtdGro, does not depend solely on the charge of the lipid^27^. Therefore, the most plausible interpretation of our binding data is lipid specificity that depends on the chemical nature of the lipid head rather than its charge.

The observed affinities of the proteins for the lipids are rather low when compared to particular known lipid-binding domains, e.g., Pleckstrin Homology (PH)^32^,^33^. These apparent dissociation constants may be related to the *in vitro* nature of the assay used, which is based on the generation of artificial membranes lacking several of the components present in an actual cellular membrane and having different lipid composition, as well as technical limitations due to poor lipid solubility^34^.

Nevertheless, several examples in the literature demonstrate that low affinity interaction have specific and important biological functions, like the glucose transporters or hexokinase and glucokinase and their different affinity for glucose^35^,^36^,^37^. Additionally, despite the apparent low K_d_ (4 mM) of Shiga toxin to its cellular receptor lipid globotriaosylceramide (GB3)^38^, the toxin enters the cells by specifically interacting with it. Also, although the affinity of Phospholipase C-ζ for particular phospholipid membranes is relatively low (molar partition coefficient K = 6 × 10^3^ M^−1^), the enzyme is anchored at the plasma membrane^39^. Additionally, the apparent dissociation constant of HspA2, a testis specific Hsp70, for the endogenous cannabinoid anandamide is ~4 μM^28^, yet HspA2 is a cytosolic carrier of this lipid. In these examples, low affinity interactions allow targeting and regulation of proteins and enzymes because only in the event of shifted equilibria specific binding would occur. Furthermore, the association of both HspA1A and HspA8 with PtdSer and the binding of the former protein with BMP have direct physiological effects^14^,^27^,^29^,^31^. Therefore, we infer that affinities with values close to the ones observed for HspA1A for PtdSer (K_d_ = 14 μM) and BMP (K_d_ = 148 μM) will be biologically important, while significantly lower affinities may not. Based on these inferences, which require experimental verification, we suggest that under the same physiological conditions: (a) only HspA1A binds to BMP and PtdOH, (b) only HspA8 binds to PtdGro, and c) both proteins bind to PtdSer, GalCer-I^3^-sulfate, and Ptd2Gro (Fig. 7).

The observed low affinities imply that high concentrations of particular lipids, e.g, PtdSer, BMP, or Ptd2Gro, or their combination are necessary for physiologically significant membrane binding of Hsp70s. Several of the lipids studied here, e.g., PtdSer, BMP, PtdOH, are particularly enriched in vesicular bodies, recycling and early endosomes, and lysosomes^40^,^41^,^42^,^43^,^44^,^45^,^46^. For example, the concentration of BMP in the late endosome is ~15 mol % of the total lipid content of the organelle and can comprise as much as 70 mol % of the lipid composition of the intraendosomal vesicles^42^,^47^. Furthermore, the concentration, localization, and saturation levels of particular lipids are altered in particular cell types (e.g., red-blood cells^48^,^49^), or because of stress (e.g., heat-shock), disease (e.g., cancer, Gaucher, Niemann-Pick), or during apoptosis^29^,^40^,^50^,^51^,^52^,^53^,^54^,^55^,^56^. These changes in membrane topography and lipid/protein ratios may facilitate the interaction of Hsp70s with lipids. The fact that Hsp70s have been observed to interact with lipids and membranes mainly in stressed, pre-apoptotic, or cancerous cells^14^,^17^,^26^,^27^,^29^,^57^,^58^,^59^,^60^,^61^ supports the above notion. Therefore, we predict that Hsp70s bind to lipids in particularly stressful conditions when enough lipid is present in specific membranes.

We further infer that the observed lipid-binding quantitative values may correlate with specific biologically important properties of HspA1A and HspA8. For example, both chaperones have been shown to promote cell death by binding externalized PtdSer at the plasma membrane of pre-apoptotic and cancerous cells^14^,^27^ and our results reveal that both HspA1A and HspA8 bind PtdSer with comparable high affinities (Table 1 and Fig. 7). Additionally, the relatively high affinity of HspA8 for PtdSer may be related with its ability to interact with PtdSer at the endosomal membrane and mediate microautophagy^31^. Given the similar affinities of the two proteins for PtdSer, however, it is not clear why HspA1A was not observed to perform the same function. We speculate that HspA1A, which is inducible and exists in minimal amounts in non-stressed cells, is not present in substantial amounts in the cell during the events of microautophagy. Furthermore, HspA1A binds to BMP and rescues the lysosomal membrane, while disruption of this interaction results in lysosomal membrane permeabilization^29^. In contrast, HspA8 fails to rescue the lysosomal membrane^29^. Given the very low affinity of HspA8 for BMP (Table 1 and Fig. 7), we suggest that this protein may not bind BMP under physiological conditions. This notion, which warrants experimental validation, might explain why HspA8 does not rescue lysosomes while HspA1A does^29^.

Promotion of cell-death, microautophagy, and lysosomal rescue are important biological processes that can be explained by the presence or absence of a specific Hsp70-lipid interaction. Furthermore, basic biological processes such as cell choice between survival and death, immune response, neurotransmission, energy homeostasis, and reproduction are controlled by the intracellular trafficking of endocannabinoids, which, is mediated by HspA2 among others^62^. We speculate that the binding of Hsp70s to lipids such as PtdOH, GalCer-I^3^-sulfate, and Ptd2Gro (Fig. 7) have physiological implications. Our overarching hypothesis, which is founded on the observed Hsp70-lipid interactions and the chemical properties of the lipids as well as their known physiological roles, states that lipid binding targets the chaperones to specific cellular sites, where, besides stabilizing proteins, they also stabilize and/or alter membranes. Hence, the Hsp70-lipid interaction provides these promiscuous protein chaperones efficient specificity to localize and function at particular membranes during cellular stress.

**Figure 1:**
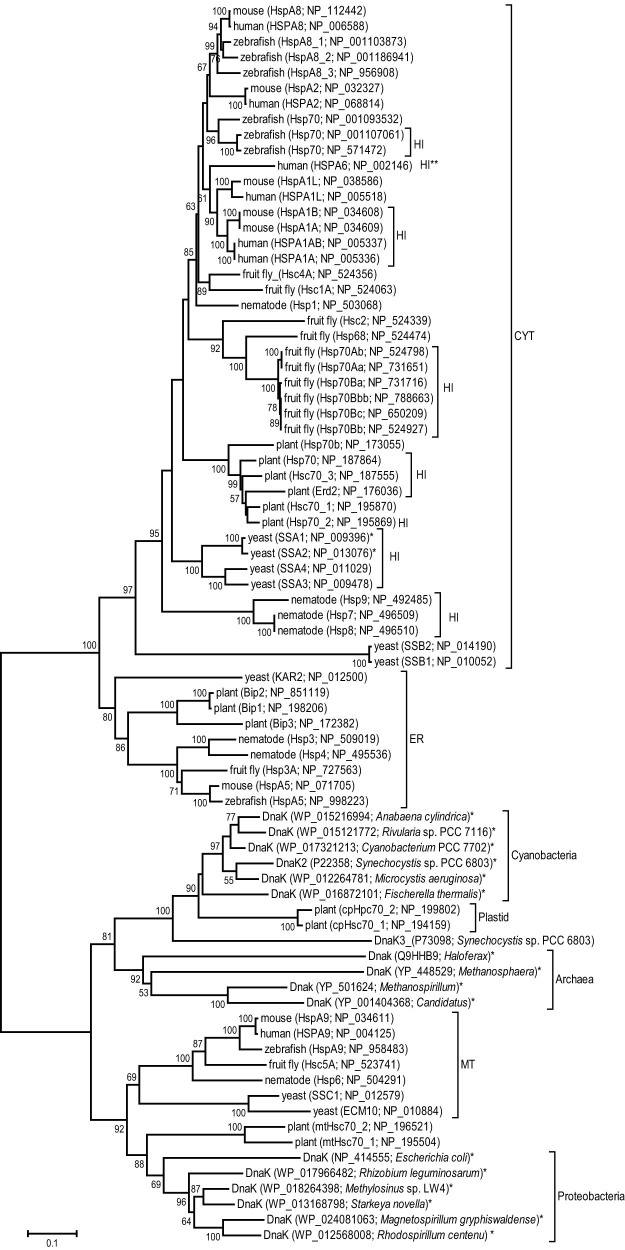
Hsp70s have diverged very early in eukaryotic evolution and their expression pattern has changed multiple times. Hsp70 protein sequences from a few representative bacterial, archaeal, and eukaryotic species were used to generate trees with two different phylogenetic methods (NJ and ML). The numbers at the nodes are bootstrap values (>50; NJ/ML). The accession numbers of the sequences used are shown next to the protein and species name. CYT: cytosolic; ER: endoplasmi reticulum; CP: chloroplast; MT: mitochondrion. Fruit fly: *Drosophila melanogaster*; nematode: *Caenorhabditis elegans*; plant: *Arabidopsis thaliana*; yeast: *Saccharomyces cerevisiae*. Heat inducible genes are denoted with HI; genes with high basal expression and heat-inducible are depicted with *; high temperature heat inducible gene present in mammals, but absent in mouse is depicted with HI**.

**Figure 2:**
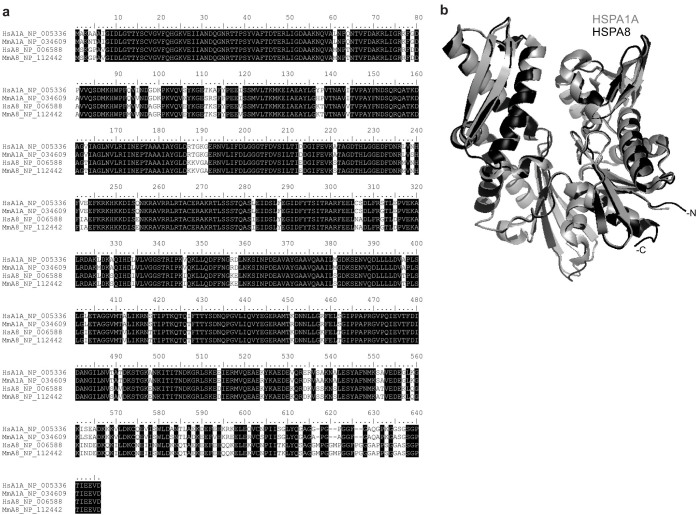
HspA1A and HspA8 have highly conserved primary sequences and are structurally identical. (a) Multiple sequence alignment of the human HspA1A and HspA8 proteins and their mice orthologs. The sequences were aligned using the MAFFT algorithm. The sequences used are: human HspA1A (HspA1A: NP_005336), mouse HspA1A (MmA1A: NP_034609), human HspA8 (HsA8: NP_006588), and mouse HspA8 (MmA8: NP_112442). The two major domains of the proteins are: the nucleotide-binding domain (NBD: amino acids 1–382) and the substrate-binding domain (amino acids 396–641). Amino acid positions 383–395 are the flexible hydrophobic linker, which connects the two domains. (b) Structural alignment between the HspA1A (grey) and HspA8 (black) NBD region showing the overall structural similarities. The structural alignment was performed using DaliLite and the figures were generated with PyMol. The PDB codes of the structures used are: 3JXU for HspA1A and 3HSC for HspA8.

**Figure 3:**
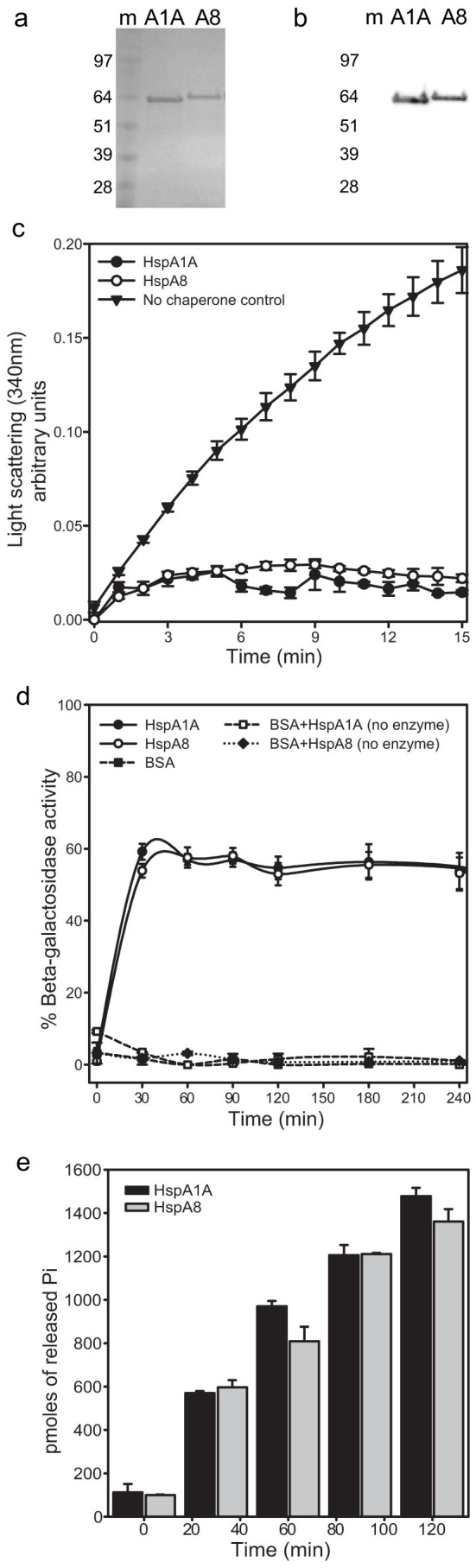
Purified recombinant HspA1A and HspA8 are functional molecular chaperones. SDS-polyacrylamide gel electrophoresis of the His-tagged HspA1A and HspA8 proteins visualized with (a) Coomassie Blue staining and (b) Western blot using a rabbit polyclonal anti-His antibody. m: molecular weight marker; A1A, HspA1A; A8, HspA8. (c) Recombinant Hsp70s suppress lysozyme aggregation. The aggregation rate was monitored by spectrophotometrically measuring the increase in turbidity as a result of protein aggregation in the presence or absence of chaperones. (d) HspA1A and HspA8 recombinant proteins refold chemically denatured β-galactosidase. The β-galactosidase assay used chemically denatured β-galactosidase and measured the recovery of the enzyme's activity by hydrolysis of ortho-nitrophenyl-β-galactoside relative to the activity of the native enzyme after addition of ATP and recombinant Hsp70s. The data are plotted as percent of activity based on the activity of the native β-galactosidase. BSA and reactions with no enzyme were used as negative controls. (e) Recombinant Hsp70s hydrolyze ATP. The ATPase assay used is a colorimetric assay that measures the amount of free inorganic phosphate (Pi). Controls that contained no chaperone or BSA were used to count for spontaneous ATP hydrolysis and their OD values were subtracted from the samples' values.

**Figure 4:**
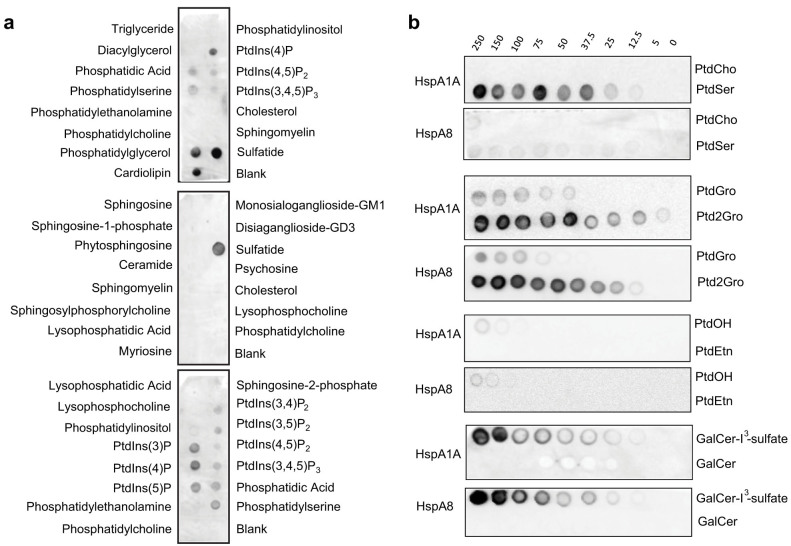
Qualitative protein-lipid overlay assay shows that recombinant HspA1A and HspA8 bind to several anionic lipids. (a) Echelon Lipid Strips using the HspA1A recombinant protein. (b) Membranes carrying serial dilutions of lipids (numbers on top in pmoles) using both proteins. Each membrane was incubated with 12 nM of each protein for 1 h at room temperature and after extensive washes, the protein that remained on the membrane because of its interaction with lipids was visualized using an anti-His antibody. Representative blots of three independent experiments using different batches of purified protein are shown.

**Figure 5:**
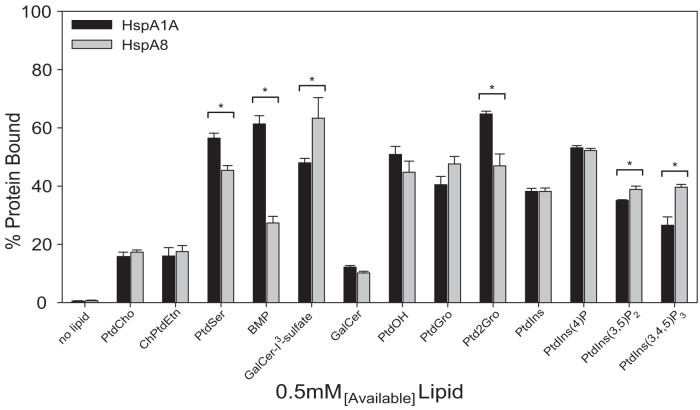
HspA1A and HspA8 binding to specific anionic lipids are quantitatively different. The vesicle sedimentation assay was performed by incubating a single concentration of liposomes (1 mM total lipid used but approximately 0.5 mM is available in the outer layer of the lipid bilayer) composed of different lipid mixtures in the specified molar ratio given in materials and methods with 1 μM of protein (for clarity only the lipid of interest is shown in the figure). After centrifugation the extent of lipid-protein binding was determined by comparing the amount of protein that remained in the supernatant to the amount of protein that was pelleted. The graphs are expressed as percentage of protein bound to lipid vesicles (Y-axis). Error bars represent standard deviations for three independent experiments. Stars denote statistical significance. The p-values obtained by comparing HspA1A and HspA8 binding values were: PtdSer, p = 0.0203; BMP, p = 0.0005; GalCer-I^3^-sulfate, p = 0.0413; Ptd2Gro, p = 0.0275; PtdIns(3,5)P_2_, p = 0.0455; PtdIns(3,4,5)P_3_, p = 0.0254.

**Figure 6:**
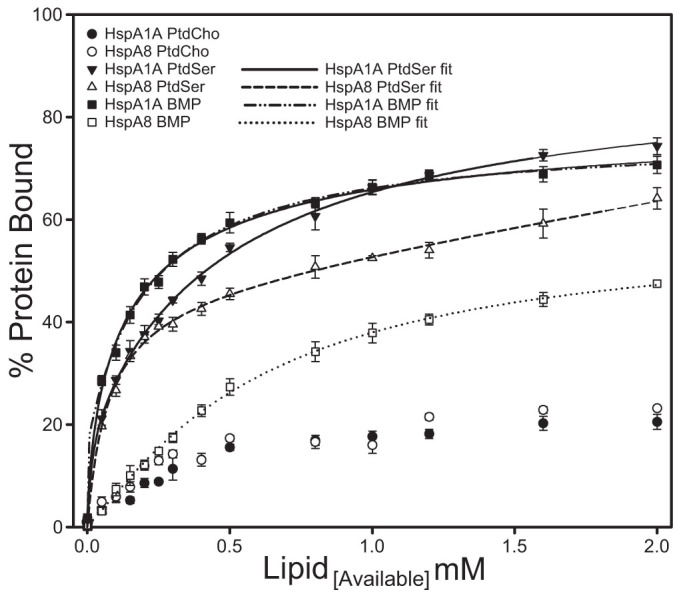
HspA1A and HspA8 binding to specific anionic lipids is quantitatively and mechanistically different. The lipid vesicle sedimentation assay was performed by incubating various concentrations of liposomes composed of different lipid mixtures in the specified molar ratio given in materials and methods with 1 μM of protein (for clarity only the lipid of interest is shown). Binding kinetics of the interaction between HspA1A and HspA8 with different lipids were determined by nonlinear least-squares analysis. Error bars represent standard errors of the calculations. Each point is the mean of three independent experiments.

**Figure 7:**
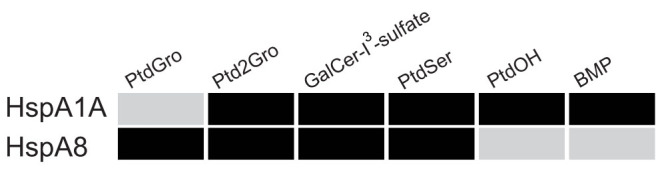
Summary of the quantitative binding results for HspA1A and HspA8 for different lipids. High binding affinities (probably physiologically relevant binding) are depicted with black filled boxes. Significantly lower affinities (probably the protein does not bind the lipid in a cell) are depicted with grey filled boxes.

The specific binding of both chaperones to particular lipids suggests that lipid binding is a conserved function of HspA1A and HspA8 since their appearance (Fig. 1). We propose that the common ancestor of these proteins would also bind to particular lipids. Although we do not know which these lipids were, we speculate that PtdSer, which both proteins bind (Table 1 and Fig. 7), is one of the lipids that their common ancestor bound. Moreover, both proteins bind to GalCer-I^3^-sulfate and Ptd2Gro. Taking into account that several bacterial Hsp70 homologs (DnaKs) bind to GalCer-I^3^-sulfate^24^, and Ptd2Gro is a lipid predominantly found in bacteria and the bacterial symbionts of eukaryotes, the mitochondria^63^, we reason that lipid-binding is an ancient function of Hsp70s conserved for several billion years. Yet, because we do not know which other lipids the bacterial Hsp70s bind, we cannot define precisely the evolution of their lipid-binding function. Furthermore, the observed Hsp70-lipid binding differences suggest that the ancient ability of these proteins has evolved. Still, it is unclear whether these changes are the result of sub- or neo-functionalization or a combination of both.

Nonetheless, our data combined with the spatially confined and differential expression of Hsp70s allow us to provide a speculative scenario for the evolution of the Hsp70-lipid binding function. Our scenario suggests a combination of sub- and neo-functionalization events^64^,^65^,^66^,^67^ and it is based on two assumptions. First, the ancestral *hsp70* genes were expressed under both normal and stress conditions like the bacterial *hsp70*, *dnaK* (Fig. 1)^68^. And second, compared to HspA1A, HspA8 is functionally and evolutionarily closer to their common ancestor, because: (a) *hspA8* has clear orthologs in all vertebrates (Fig. 1), while *hspA1A* does not^6^,^8^; (b) *hspA1A* is heat inducible and inducibility has evolved independently several times (Fig. 1); and (c), HspA8 binds to PtdGro (Fig. 7), the predominant bacterial lipid, while HspA1A does not.

According to our scenario, the coding sequence of the ancestral *hsp70* genes diverged via nucleotide substitutions very early during eukaryotic evolution, because spatially confined Hsp70s form deep phylogenetic clades (Fig. 1). This relatively rapid sequence divergence, sub-functionalization^6^,^64^,^65^, introduced signal peptides and retention motifs that targeted and limited the proteins at particular subcellular compartments resulting in specialized spatial expression patterns. Additionally, during the course of their evolution *hsp70* genes acquired neutral mutations in their promoter regions; some copies retained part of the ancestral expression pattern whereas others did not resulting in either constitutively expressed or heat-induced genes (Fig. 1)^6^,^64^,^65^. Despite the divergence in both coding and non-coding gene sequences, the amino acid sites responsible for the protein chaperone function were retained by strong purifying selection. However, the sites responsible for lipid-binding mutated, reached fixation, and ensued biochemical specialization and functional differentiation. Such sequence divergence most probably concurred with the evolution of membrane lipid composition, which changed dramatically during the transition from prokaryotes to eukaryotes as well as between the different eukaryotic membranes^69^,^70^. The ancestral Hsp70 proteins were able to bind lipids common in bacterial membranes like PtdGro, Ptd2Gro, and GalCer-I^3^-sulfate. Sub-functionalization limited (some of) these functions to specific *hsp70* copies. For example HspA8 retained PtdGro-binding whereas HspA1A did not (Fig. 7). This sub-functionalization was followed by prolonged neo-functionalization^64^ resulting in binding to new lipid ligands, for example BMP. The neo-functionalizing mutations were most probably adaptive because they must have affected cell survival.

The differential lipid-binding profiles of the highly conserved and almost identical molecular chaperones HspA1A and HspA8 reveal that sequence divergence via single amino acid replacements led to highly specialized functions, which directly affected cellular homeostasis. In this report we define an evolutionary path, where promiscuous protein-protein interactions are combined with specialized protein-lipid interactions to coordinate the ability of cells to cope with stress and survive in an ever-changing environment.

## Methods

### Lipids, Chemicals, and Reagents

Phosphatidylcholine [PtdCho; 1,2-dipalmitoyl-sn-glycero-3-phosphocholine], Phosphatidylethanolamine [PtdEtn; 1,2-dihexadecanoyl-*sn*-glycero-3-phosphoethanolamine], Phosphatidylserine [PtdSer; 1,2-dipalmitoyl-sn-glycero-3-phosphoserine (sodium salt)], Phosphatidic acid [PtdOH; 1,2-dipalmitoyl-sn-glycero-3-phosphate (sodium salt)], Phosphatidylglycerol [PtdGro; 1,2-dipalmitoyl-sn-glycero-3-phosphoglycerol (sodium salt)], Phosphatidylinositol [PtdIns; 1,2-dipalmitoyl-sn-glycero-3-phospho-(1′-myo-inositol) (ammonium salt)], Phosphatidylinositol 4-phosphate [PtdIns(4)P; 1,2-dioleoyl-sn-glycero-3-phospho-(1′-myo-inositol-4′-phosphate) (ammonium salt)], Phosphatidylinositol 3,5-bisphosphate [PtdIns(3,5)P_2_; 1,2-dioleoyl-sn-glycero-3-phospho-(1′-myo-inositol-3′,5′-bisphosphate) (ammonium salt)], and Phosphatidylinositol (3,4,5)-triphosphate [PtdIns(3,4,5)P_3_; 1,2-dioleoyl-sn-glycero-3-phospho-(1′-myo-inositol-3′,4′,5′-trisphosphate) (ammonium salt)], Cardiolipin [Ptd2Gro; 1,1′,2,2′-tetramyristoyl cardiolipin (ammonium salt)], Galactosyl Ceramide [GalCer; D-galactosyl-ß-1,1′ N-palmitoyl-D-*erythro*-sphingosine], Sulfatide [GalCer-I^3^-sulfate; 3-O-Sulfo-D-Galactosyl-ß1-1′-N-Lignoceroyl-D-erythro-Sphingosine (ammonium salt)], bis(monoacylglycerol)phosphate [BMP; sn-(3-oleoyl-2-hydroxy)-glycerol-1-phospho-sn-3′-(1′-oleoyl-2′-hydroxy)-glycerol (ammonium salt)], and Cholesterol [Ch; (3β)-cholest-5-en-3-ol)], were purchased from Avanti Polar Lipids, Inc. (Alabaster, AL) and Echelon Biosciences Inc. Salt Lake City, UT). Other common chemicals and reagents, e.g., antibiotics, buffers, and growth media, were obtained from Fisher Scientific or Sigma-Aldrich (St. Louis, MO).

### Protein sequence analysis and phylogeny

Protein sequences were collected from the National Center for Biotechnology Information (NCBI) protein databank using either keyword or protein-BLAST^71^ searches with the human HspA1A and HspA8 as queries and default parameters. The sequences were aligned with MAFFT^72^ using the E-INS-i strategy and default parameters, and manually corrected with BioEdit. Maximum-likelihood (ML) was used to find the best model of evolution (MEGA 6.0)^73^. Based on the Bayesian Information Criterion (BIC) the substitution pattern was best described by the General Reverse Transcriptase (rtREV) model with corrections for non-uniform evolutionary rates among sites (+G; gamma value = 1.099), and by assuming that a certain fraction of sites are evolutionarily invariable (+I; 13.17% of sites). Phylogenetic trees were generated using the neighbor-joining (NJ) and ML algorithms as implemented in MEGA 6.0^73^. One thousand bootstrap pseudo-replicates were used to test the reliability of the inferred trees. All positions containing gaps were eliminated and there were a total of 559 positions in the final dataset.

Protein structural data were collected from the Protein Database (PDB) and were aligned using the DaliLite tool^74^. The electrostatic potential of the protein surfaces was calculated by solving the Poisson-Boltzmann equation using the PBEQ Solver tool^75^. Structural figures were generated using Pymol (Delano Scientific).

### Generation of Recombinant DNA Clones

The mouse cDNA clones containing the *hspA1A* and *hspA8* gene sequences, accession numbers BC054782 and BC089457 respectively, were purchased from OpenBiosystems (GE Dharmacon). Clones corresponding to the HspA1A and HspA8 full-length gene were generated by the polymerase chain reaction (PCR) method using specific oligonucleotide primers. The primer sequences used as well as the restriction enzymes (NdeI and XhoI; underlined nucleotides) that were incorporated for directional cloning of the genes were: CCGCATATGATGGCCAAGAACACGGCG and CAGCTCGAGATCCACCTCCTCGATGGT for *hspA1A*, and CCGCATATGATGTCTAAGGGACCTGCA and CAGCTCGAGATCCACCTCTTCAATGGT for *hspA8*.

The amplified DNA fragments were then cloned into the protein expression vector pET-22b+ (Novagen) using the Rapid DNA Ligation Kit (Roche) following the manufacturer's protocol. The ligation mixtures were later transformed in *Escherichia coli* strain DH5α cells (Life Technologies), the positive colonies were verified by PCR, and the intact open reading frames were verified by DNA sequencing.

### Generation and Purification of Recombinant Proteins

Purified plasmid DNA of sequence-verified recombinant clones was subsequently transformed into BL21(DE3) *E. coli* cells (Life Technologies). A single colony was then added to 15 mL of Luria-Bertani (LB) broth with Ampicilin (100 μg/mL) and grown until an OD of between 0.8 and 1.0 was reached. Recombinant protein production was induced using 1 mM (final concentration) of Isopropyl β-D-1-thiogalactopyranoside (IPTG) at 25°C for 14–16 h. The cultures were pelleted by centrifugation and the cells were lysed in a lysis buffer containing 50 mM sodium phosphate, pH 7.4, and 300 mM sodium chloride. During lysis, Phenylmethylsulfonyl fluoride (PMSF) (1 mM), lysozyme (0.5 mg/mL), and Triton-X (1%) were added, and the lysates were sonicated until optically clear. After sonication, the lysates were rotated at 4°C for 30 min and were centrifuged at 10,000 × g for 5′. The supernatant, containing the soluble Hsp70 proteins, was mixed with Cobalt agarose beads (Pierce), equilibrated in the same buffer and rotated, at 4°C for 1 h. The samples were then centrifuged at 700 × g for 2 min and the beads were washed 3× with the same buffer to remove proteins that did not interact with the cobalt beads. Finally, the recombinant proteins were eluted from the beads by incubation with equal volume of lysis buffer containing 150 mM imidazole. The elutions were then dialyzed extensively against a 25 mM Tris-HCl or 25 mM HEPES, pH 7.4, buffer using Amicon Ultra centrifugal filters.

The protein concentration was determined using the Coomassie Blue Plus Protein Assay Reagent (Pierce) following the protocol supplied by the manufacturer. The concentration (μg/μl) values were transformed to moles/μl by dividing them with the protein molecular weight. Protein purity was assessed by sodium dodecyl sulfate polyacrylamide gel electrophoresis (SDS-PAGE). Separated proteins were detected by staining with Simply Blue™ Safe Stain (Life Sciences). For Western blotting, separated proteins were transferred to nitrocellulose (Protran; Whatman) and blocked with 5% milk powder, in 50 mM Tris-HCl, pH 7.4, 150 mM NaCl, and 0.05% Tween 20 (TBST) for 1 h at room temperature. Western blots were probed with a polyclonal anti-His antibody (Thermo Scientific; 1:2000 in TBST) overnight at 4°C. The secondary antibody, peroxidase-conjugated goat anti-rabbit immunoglobulin (Thermo Scientific; 1:10000 in TBST), was incubated with the nitrocellulose for 1 h at room temperature. Bound antibody was visualized with the Pierce ECL Western Blotting Substrate (Pierce). All gels and signals were collected using the Omega Lum C system from Aplegen.

### Protein Activity Tests

To test whether the recombinant proteins were functional molecular chaperones it was determined whether they (i) suppress protein aggregation, (ii) refold denatured proteins, and (iii) hydrolyze ATP. All experiments were repeated at least three times using different batches of protein.

- The aggregation assay included the chemical denaturation of lysozyme by incubating 10 mg/ml of lysozyme at 40°C for 2 hours into a buffer containing 50 mM Tris-HCl, pH 8, 8 M Urea, 10 mM DTT, and 2 mM EDTA^76^. The denatured enzyme was then diluted 100 fold in a non-denaturing buffer (50 mM Tris-HCl, pH 8, 80 mM Urea, and 1 mM EDTA, which resulted in lysozyme precipitation. The aggregation rate was then monitored by measuring the increase in light scattering (turbidity) as a result of protein aggregation spectrophotometrically at 340 nM every minute for 15 min. Addition of 2 μM HspA1A and HspA8 prevented the turbidity of the solution to increase, showing that the latter proteins prevent protein aggregation. Control reactions containing no chaperones were used.
- The β-galactosidase refolding assay^77^ used chemically denatured β-galactosidase and measured the recovery of the enzyme's activity by hydrolysis of the ortho-Nitrophenyl-β-galactoside relative to the activity of the native enzyme after addition of ATP and recombinant Hsp70s. BSA and reactions with no enzyme were used as negative controls. Specifically, β-galactosidase was added to either Glycylglycine (native buffer) or to unfolding buffer (25 mM HEPES, pH 7.4, 5 mM MgCl_2_, 50 mM KCl, 2 mM ATP, 10 mM DTT). The enzyme was incubated at 30°C for 30 min. The reactions contained the control protein BSA (3.2 μM) or Hsp70 (2 μM) and β-galactosidase (native or denatured) to a final volume of 124 μl were incubated at 37°C for the duration of the experiment. Samples were taken from each reaction every 30 minute for a total of 240 min, added to ONPG, and incubated at 37°C for 15 min. These enzymatic reactions were stopped by the addition of 0.5 M sodium carbonate. The absorbance was read at 450 nM and the percent activity of the β-galactosidase was determined using the following equation: % activity = 100*(test-absorbance/native-absorbance). Percent activity was plotted versus time to compare the amount of β-galactosidase that recovered its function, which correlates with the amount of β-galactosidase refolded by the chaperone.
- The ATPase assay used is a colorimetric assay that measures the amount of free inorganic phosphate. This assay was performed by incubating 1 μM of recombinant Hsp70 with 4 mM ATP at 37°C for 120 minutes and the release of inorganic phosphate (Pi) was quantified every 30 min using the colorimetric assay QuantiChrom™ ATPase/GTPase Assay kit (BioAssay Systems). To determine the amount of Pi released a standard curve was generated by measuring the absorbance produced by known Pi concentrations. Controls that contained no chaperone or BSA were also used to count for spontaneous ATP hydrolysis. The final amounts of Pi released were calculated by subtracting the control values from each sample.

### Lipid Binding Assays

The verified for functionality proteins were then tested for their ability to bind to lipids using the well-established protein-lipid overlay (PLO) and lipid vesicle sedimentation (LVS) assays.

The PLO assay^78^ served as a first screen to determine the spectrum of lipids that the two proteins bind, because the results of these assays cannot be quantified accurately and reliably^34^. Using this assay as implemented by Echelon Lipid Strips™ we first screened HspA1A and identified several candidate lipids that were further explored using additional tests. Although the Echelon assay represents a fast and easy way to identify lipid ligands, it contains a fixed amount of lipids and a limited array of lipids. Therefore, we also generated lipid-bearing nitrocellulose membranes employing the method described by Dowler et al. (2002). Specifically, lipids were diluted in a 1:1 mix of chloroform and methanol to reach the desired concentrations ranging from 0 to 250 picomoles. In the dark, 1 μl of each concentration of the lipids was spotted in a single row onto a strip of nitrocellulose membrane (Hybond-C Extra; Amersham Biosciences). The strips were allowed to air dry for 1 h in a dark box. After drying, the strips were blocked for 1 hour using 3% fat free BSA in TBST. After blocking of the membrane, 12 nM of HspA1A or HspA8 were incubated with the strips for 1 h. The lipid strips were washed with TBST and the protein remained on the strip, because of its interaction with a particular lipid, was detected using the western blot procedure described above. All experiments were repeated at least three times using different batches of protein.

To quantify the lipid binding of HspA1A and HspA8 the LVS^34^ assay was used. This method involves the quantitative pelleting of liposomal vesicles from a lipid/protein mixture. The extent of lipid-protein binding was then evaluated by comparing the amount of protein that remained in the supernatant to the amount of protein that was pelleted. The assay requires that the protein does not precipitate and its precipitation is not promoted by the presence of the lipid due to non-specific binding. Therefore, we used proteins that do not precipitate and several control-lipids^34^. Control lipids are lipids that have similar or identical hydrophobic tails to lipids that Hsp70s bind, but have different head groups and do not bind or show minimal binding to HspA1A or HspA8 (for example PtdCho and GalCer are control lipids for PtdSer and GalCer-I^3^-sulfate).

In all experiments PtdCho, the major constituent of eukaryotic plasma membranes, showed very low basal binding to both proteins and thus it was used as a backbone lipid. Next, liposomal vesicles that resemble physiological membranes were generated by mixing PtdCho with the lipid of interest in molar ratios that parallel those in cellular membranes^46^. The lipid mixtures tested and their mol/mol ratios (given in parenthesis) were: PtdCho:PtdSer (80:20), PtdCho:Ch:PtdEtn (45:35:20), PtdCho:PtdOH (90:10), PtdCho:PtdGro (90:10), PtdCho:PtdIns (95:5), PtdCho:PtdIns(4)P (95:5), PtdCho:PtdIns(3,5)P2 (95:5), PtdCho:PtdIns(3,4,5)P3 (95:5), PtdCho:Ptd2Gro (85:15), PtdCho:GalCer (90:10), PtdCho:GalCer-I^3^-sulfate (90:10), PtdCho:BMP (70:30). These mixtures were dried under vacuum for approximately 40 min and then were hydrated at room temperature for 1 hour in a buffer containing 25 mM HEPES, pH 7.4, and 100 mM NaCl (HBS) and vortexed frequently. Within 30 min of hydration, the samples were probe-sonicated for 10 × 1 second bursts. After 1 hour hydration, multilayered vesicles were formed. To generate the final uniform population of small, unilamellar vesicles, the samples were subjected to 8 cycles of freezing in liquid nitrogen and thawing in a bath sonicator (45°C, 10 minute sonics) until optically clear. The binding reactions contained a fixed concentration (1 μM) of Hsp70 in a total reaction volume of 100 μl. A specific concentration of lipid vesicles was added and the reactions were incubated at 30°C for 30 min.

After the incubation period, the samples were transferred to ultracentrifuge tubes and ultracentrifuged at 166,000 × g at 25°C for 40 min. After centrifugation, the supernatant was removed and saved in microcentrifuge tubes. The pellets were then resuspended in equal volume of HBS. Equal volumes of supernatant and pellet fractions, which contained unbound and bound to liposomes proteins, respectively, were separated on an SDS-PAGE as described above. The gels were stained as described above and relative protein amounts were quantified by densitometry using the Omega Lum C (Aplegen) software. All experiments were repeated at least three times using different batches of protein.

### Binding affinities

To determine the affinity of HspA1A and HspA8 to various lipids the LVS assay was performed using increasing concentrations of vesicles (0.1–4 mM) and a fixed concentration (1 μM) of protein. The reactions were incubated and processed exactly as described above. All experiments were repeated with at least three different batches of protein. SigmaPlot (version 10.0, Systat Software Inc) was used to graph all lipid vesicle sedimentation assay data and to fit the binding data to various equations corresponding to specific binding models.

### Statistical tests

Non-linear regression analysis as implemented in SigmaPlot (version 10.0, Systat Software Inc) was used to determine the goodness of fit of the data to a particular theoretical binding model. The same program was also used to calculate mean binding and standard deviations. Statistical significance was determined by an unpaired t-test. A P value < 0.05 was considered statistically significant.

## Author Contributions

C.M. and N.N. designed the study. C.M., M.C.S., F.S. and N.N. performed the experiments. C.M., D.C. and N.N. analyzed the data and performed statistical analysis. C.M., D.C. and N.N. wrote the manuscript. The contents are the responsibility of the authors and do not necessarily reflect the views of USAID or the United States Government.

## Supplementary Material

Supplementary Information Supplementary Data

## Figures and Tables

**Table 1:** Binding kinetics of HspA1A (A1A) and HspA8 (A8) to PtdSer and BMP

	PtdSer		BMP	
	A1A	A8	A1A	A8
K_d1_	14 ± 1.9	72 ± 8.3	148 ± 19.1	598 ± 73.2
K_d2_	520 ± 141			
B_max1_	19.6 ± 7.5	46.4 ± 1.6	79.6 ± 3.7	57.9 ± 4.2
B_max2_	70.1 ± 5.2			
a	ΝΑ	NA	0.7 ± 0.1	1.2 ± 0.1
B_0_	ΝΑ	NA	1.98 ± 1.3	0.4 ± 0.1
N_s_	ΝΑ	9.4 ± 0.9	NA	NA

K_d_ (apparent dissociation constant), μM; B_max_ (maximal binding), % protein bound; a (Hill coefficient); B_0_, initial binding; N_s_, non-specific binding.

## References

[b1] Kultz D. Molecular and evolutionary basis of the cellular stress response. Annu Rev Physiol 67, 225–257 (2005). 15709958 10.1146/annurev.physiol.67.040403.103635

[b2] Kultz D. Evolution of the cellular stress proteome: from monophyletic origin to ubiquitous function. J Exp Biol 206, 3119–3124 (2003). 12909693 10.1242/jeb.00549

[b3] Lindquist S. & Craig E. A. The heat-shock proteins. Annu Rev Genet 22, 631–677 (1988). 2853609 10.1146/annurev.ge.22.120188.003215

[b4] Gupta R. S. & Singh B. Phylogenetic analysis of 70 kD heat shock protein sequences suggests a chimeric origin for the eukaryotic cell nucleus. Curr Biol 4, 1104–1114 (1994). 7704574 10.1016/s0960-9822(00)00249-9

[b5] Kabani M. & Martineau C. N. Multiple hsp70 isoforms in the eukaryotic cytosol: mere redundancy or functional specificity? Curr Genomics 9, 338–248 (2008). 19471609 10.2174/138920208785133280 PMC2685646

[b6] Brocchieri L., Conway de Macario E. & Macario A. hsp70 genes in the human genome: Conservation and differentiation patterns predict a wide array of overlapping and specialized functions. BMC Evol Biol 8, 19 (2008). 18215318 10.1186/1471-2148-8-19 PMC2266713

[b7] Kampinga H. H. & Craig E. A. The HSP70 chaperone machinery: J proteins as drivers of functional specificity. Nat Rev Mol Cell Biol 11, 579–592 (2010). 20651708 10.1038/nrm2941 PMC3003299

[b8] Kourtidis A. *et al.* Identification of several cytoplasmic HSP70 genes from the Mediterranean mussel (Mytilus galloprovincialis) and their long-term evolution in Mollusca and Metazoa. J Mol Evol 62, 446–459 (2006). 16547643 10.1007/s00239-005-0121-4

[b9] Nikolaidis N. & Nei M. Concerted and nonconcerted evolution of the Hsp70 gene superfamily in two sibling species of nematodes. Mol Biol Evol 21, 498–505 (2004). 14694072 10.1093/molbev/msh041

[b10] Krenek S., Schlegel M. & Berendonk T. U. Convergent evolution of heat-inducibility during subfunctionalization of the Hsp70 gene family. BMC Evol Biol 13, 49 (2013). 23433225 10.1186/1471-2148-13-49 PMC3606833

[b11] James P., Pfund C. & Craig E. A. Functional specificity among Hsp70 molecular chaperones. Science 275, 387–389 (1997). 8994035 10.1126/science.275.5298.387

[b12] Pfund C., Huang P., Lopez-Hoyo N. & Craig E. A. Divergent functional properties of the ribosome-associated molecular chaperone Ssb compared with other Hsp70s. Mol Biol Cell 12, 3773–3782 (2001). 11739779 10.1091/mbc.12.12.3773 PMC60754

[b13] Knight S. A., Sepuri N. B., Pain D. & Dancis A. Mt-Hsp70 homolog, Ssc2p, required for maturation of yeast frataxin and mitochondrial iron homeostasis. J Biol Chem 273, 18389–18393 (1998). 9660806 10.1074/jbc.273.29.18389

[b14] Arispe N., Doh M., Simakova O., Kurganov B. & De Maio A. Hsc70 and Hsp70 interact with phosphatidylserine on the surface of PC12 cells resulting in a decrease of viability. FASEB J 18, 1636–1645 (2004). 15522909 10.1096/fj.04-2088com

[b15] Calderwood S. K., Mambula S. S. & Gray P. J. Jr Extracellular heat shock proteins in cell signaling and immunity. Ann N Y Acad Sci 1113, 28–39 (2007). 17978280 10.1196/annals.1391.019

[b16] De Maio A. Extracellular heat shock proteins, cellular export vesicles, and the Stress Observation System: a form of communication during injury, infection, and cell damage. It is never known how far a controversial finding will go! Dedicated to Ferruccio Ritossa. Cell Stress Chaperon 16, 235–249 (2011). 10.1007/s12192-010-0236-4 PMC3077223 20963644

[b17] De Maio A. Extracellular Hsp70: export and function. Curr Protein Pept Sci 15, 225–231 (2014). 24694368 10.2174/1389203715666140331113057

[b18] Guidon P. T. Jr & Hightower L. E. The 73 kilodalton heat shock cognate protein purified from rat brain contains nonesterified palmitic and stearic acids. J Cell Physiol 128, 239–245 (1986). 3733888 10.1002/jcp.1041280215

[b19] Guidon P. T. Jr & Hightower L. E. Purification and initial characterization of the 71-kilodalton rat heat-shock protein and its cognate as fatty acid binding proteins. Biochemistry 25, 3231–3239 (1986). 3730359 10.1021/bi00359a023

[b20] Horvath I., Multhoff G., Sonnleitner A. & Vigh L. Membrane-associated stress proteins: more than simply chaperones. Biochim Biophys Acta 1778, 1653–1664 (2008). 18371297 10.1016/j.bbamem.2008.02.012

[b21] Lancaster G. I. & Febbraio M. A. Mechanisms of stress-induced cellular HSP72 release: implications for exercise-induced increases in extracellular HSP72. Exerc Immunol Rev 11, 46–52 (2005). 16385843

[b22] Mambula S. S., Stevenson M. A., Ogawa K. & Calderwood S. K. Mechanisms for Hsp70 secretion: crossing membranes without a leader. Methods 43, 168–175 (2007). 17920512 10.1016/j.ymeth.2007.06.009 PMC2745244

[b23] Mamelak D. & Lingwood C. The ATPase domain of hsp70 possesses a unique binding specificity for 3′-sulfogalactolipids. J Biol Chem 276, 449–456 (2001). 11024054 10.1074/jbc.M006732200

[b24] Mamelak D. *et al.* Hsp70s contain a specific sulfogalactolipid binding site. Differential aglycone influence on sulfogalactosyl ceramide binding by recombinant prokaryotic and eukaryotic hsp70 family members. Biochemistry 40, 3572–3582 (2001). 11297423 10.1021/bi001643u

[b25] Misra U. K., Deedwania R. & Pizzo S. V. Binding of activated alpha2-macroglobulin to its cell surface receptor GRP78 in 1-LN prostate cancer cells regulates PAK-2-dependent activation of LIMK. J Biol Chem 280, 26278–26286 (2005). 15908432 10.1074/jbc.M414467200 PMC1201553

[b26] Multhoff G. Heat shock proteins in immunity. In: Handb Exp Pharmacol (ed^^∧^^(eds) (2006). 10.1007/3-540-29717-0_12 16610364

[b27] Schilling D. *et al.* Binding of heat shock protein 70 to extracellular phosphatidylserine promotes killing of normoxic and hypoxic tumor cells. FASEB J 23, 2467–2477 (2009). 19289606 10.1096/fj.08-125229 PMC2717766

[b28] Oddi S. *et al.* Molecular identification of albumin and Hsp70 as cytosolic anandamide-binding proteins. Chem Biol 16, 624–632 (2009). 19481477 10.1016/j.chembiol.2009.05.004

[b29] Kirkegaard T. *et al.* Hsp70 stabilizes lysosomes and reverts Niemann-Pick disease-associated lysosomal pathology. Nature 463, 549–553 (2010). 20111001 10.1038/nature08710

[b30] Arispe N., Doh M. & De Maio A. Lipid interaction differentiates the constitutive and stress-induced heat shock proteins Hsc70 and Hsp70. Cell Stress Chaperon 7, 330–338 (2002). 10.1379/1466-1268(2002)007<0330:lidtca>2.0.co;2 PMC514832 12653477

[b31] Sahu R. *et al.* Microautophagy of cytosolic proteins by late endosomes. Dev Cell 20, 131–139 (2011). 21238931 10.1016/j.devcel.2010.12.003 PMC3025279

[b32] Lemmon M. A. Pleckstrin homology (PH) domains and phosphoinositides. Biochem Soc Symp 74, 81–93 (2007). 17233582 10.1042/BSS0740081 PMC3777418

[b33] Stahelin R. V. Lipid binding domains: more than simple lipid effectors. J Lipid Res 50 **Suppl**, S299–304 (2009). 19008549 10.1194/jlr.R800078-JLR200 PMC2674730

[b34] Narayan K. & Lemmon M. A. Determining selectivity of phosphoinositide-binding domains. Methods 39, 122–133 (2006). 16829131 10.1016/j.ymeth.2006.05.006 PMC3786563

[b35] Becker T. C. *et al.* Differential effects of overexpressed glucokinase and hexokinase I in isolated islets. Evidence for functional segregation of the high and low Km enzymes. J Biol Chem 271, 390–394 (1996). 8550593 10.1074/jbc.271.1.390

[b36] Irwin D. M. & Tan H. Evolution of glucose utilization: glucokinase and glucokinase regulator protein. Mol Phylogenet Evol 70, 195–203 (2014). 24075984 10.1016/j.ympev.2013.09.016 PMC3897444

[b37] Wilson J. E. Isozymes of mammalian hexokinase: structure, subcellular localization and metabolic function. J Exp Biol 206, 2049–2057 (2003). 12756287 10.1242/jeb.00241

[b38] Gallegos K. M. *et al.* Shiga toxin binding to glycolipids and glycans. PLoS One 7, e30368 (2012). 22348006 10.1371/journal.pone.0030368 PMC3278406

[b39] Nomikos M. *et al.* Binding of phosphoinositide-specific phospholipase C-zeta (PLC-zeta) to phospholipid membranes: potential role of an unstructured cluster of basic residues. J Biol Chem 282, 16644–16653 (2007). 17430887 10.1074/jbc.M701072200

[b40] Andreyev A. Y. *et al.* Subcellular organelle lipidomics in TLR-4-activated macrophages. J Lipid Res 51, 2785–2797 (2010). 20574076 10.1194/jlr.M008748 PMC2918461

[b41] Connor J., Bucana C., Fidler I. J. & Schroit A. J. Differentiation-dependent expression of phosphatidylserine in mammalian plasma membranes: quantitative assessment of outer-leaflet lipid by prothrombinase complex formation. Proc Natl Acad Sci U S A 86, 3184–3188 (1989). 2717615 10.1073/pnas.86.9.3184 PMC287091

[b42] Hullin-Matsuda F., Taguchi T., Greimel P. & Kobayashi T. Lipid compartmentalization in the endosome system. Semin Cell Dev Biol 31, 48–56 (2014). 24747366 10.1016/j.semcdb.2014.04.010

[b43] Slaughter B. D. *et al.* Non-uniform membrane diffusion enables steady-state cell polarization via vesicular trafficking. Nat Commun 4, 1380 (2013). 23340420 10.1038/ncomms2370 PMC3900288

[b44] Uchida Y. *et al.* Intracellular phosphatidylserine is essential for retrograde membrane traffic through endosomes. Proc Natl Acad Sci U S A 108, 15846–15851 (2011). 21911378 10.1073/pnas.1109101108 PMC3179068

[b45] van Meer G. & de Kroon A. I. Lipid map of the mammalian cell. J Cell Sci 124, 5–8 (2011). 21172818 10.1242/jcs.071233

[b46] van Meer G., Voelker D. R. & Feigenson G. W. Membrane lipids: where they are and how they behave. Nat Rev Mol Cell Biol 9, 112–124 (2008). 18216768 10.1038/nrm2330 PMC2642958

[b47] Kobayashi T. *et al.* Separation and characterization of late endosomal membrane domains. J Biol Chem 277, 32157–32164 (2002). 12065580 10.1074/jbc.M202838200

[b48] Boas F. E., Forman L. & Beutler E. Phosphatidylserine exposure and red cell viability in red cell aging and in hemolytic anemia. Proc Natl Acad Sci U S A 95, 3077–3081 (1998). 9501218 10.1073/pnas.95.6.3077 PMC19697

[b49] Yasin Z. *et al.* Phosphatidylserine externalization in sickle red blood cells: associations with cell age, density, and hemoglobin F. Blood 102, 365–370 (2003). 12609840 10.1182/blood-2002-11-3416

[b50] Balogh G. *et al.* Lipidomics reveals membrane lipid remodelling and release of potential lipid mediators during early stress responses in a murine melanoma cell line. Biochim Biophys Acta 1801, 1036–1047 (2010). 20430110 10.1016/j.bbalip.2010.04.011

[b51] Haak J. L., Buettner G. R., Spitz D. R. & Kregel K. C. Aging augments mitochondrial susceptibility to heat stress. Am J Physiol Regul Integr Comp Physiol 296, R812–820 (2009). 19144753 10.1152/ajpregu.90708.2008 PMC2665848

[b52] Petersen N. H., Kirkegaard T., Olsen O. D. & Jaattela M. Connecting Hsp70, sphingolipid metabolism and lysosomal stability. Cell cycle (Georgetown, Tex 9 (2010). 10.4161/cc.9.12.12052 20519957

[b53] Pineau L. *et al.* Lipid-induced ER stress: synergistic effects of sterols and saturated fatty acids. Traffic 10, 673–690 (2009). 19302420 10.1111/j.1600-0854.2009.00903.x

[b54] Zha B. S. & Zhou H. ER Stress and Lipid Metabolism in Adipocytes. Biochem Res Int 2012, 312943 (2012). 22400114 10.1155/2012/312943 PMC3287011

[b55] Hein L. K., Duplock S. & Fuller M. Selective reduction of bis(monoacylglycero)phosphate ameliorates the storage burden in a THP-1 macrophage model of Gaucher disease. J Lipid Res 54, 1691–1697 (2013). 23564732 10.1194/jlr.M038232 PMC3646469

[b56] Hein L. K., Duplock S., Hopwood J. J. & Fuller M. Lipid composition of microdomains is altered in a cell model of Gaucher disease. J Lipid Res 49, 1725–1734 (2008). 18427156 10.1194/jlr.M800092-JLR200 PMC2444005

[b57] Arap M. A. *et al.* Cell surface expression of the stress response chaperone GRP78 enables tumor targeting by circulating ligands. Cancer Cell 6, 275–284 (2004). 15380518 10.1016/j.ccr.2004.08.018

[b58] Gastpar R. *et al.* Heat shock protein 70 surface-positive tumor exosomes stimulate migratory and cytolytic activity of natural killer cells. Cancer Res 65, 5238–5247 (2005). 15958569 10.1158/0008-5472.CAN-04-3804 PMC1785299

[b59] Gehrmann M. *et al.* Tumor-specific Hsp70 plasma membrane localization is enabled by the glycosphingolipid Gb3. PLoS ONE 3, e1925 (2008). 18382692 10.1371/journal.pone.0001925 PMC2271151

[b60] Shin B. K. *et al.* Global profiling of the cell surface proteome of cancer cells uncovers an abundance of proteins with chaperone function. J Biol Chem 278, 7607–7616 (2003). 12493773 10.1074/jbc.M210455200

[b61] Vega V. L. *et al.* Hsp70 translocates into the plasma membrane after stress and is released into the extracellular environment in a membrane-associated form that activates macrophages. J Immunol 180, 4299–4307 (2008). 18322243 10.4049/jimmunol.180.6.4299

[b62] Maccarrone M., Dainese E. & Oddi S. Intracellular trafficking of anandamide: new concepts for signaling. Trends Biochem Sci 35, 601–608 (2010). 20570522 10.1016/j.tibs.2010.05.008

[b63] Haines T. H. A new look at Cardiolipin. Biochim Biophys Acta 1788, 1997–2002 (2009). 19801076 10.1016/j.bbamem.2009.09.008

[b64] He X. & Zhang J. Rapid subfunctionalization accompanied by prolonged and substantial neofunctionalization in duplicate gene evolution. Genetics 169, 1157–1164 (2005). 15654095 10.1534/genetics.104.037051 PMC1449125

[b65] Lynch M. & Force A. The probability of duplicate gene preservation by subfunctionalization. Genetics 154, 459–473 (2000). 10629003 10.1093/genetics/154.1.459 PMC1460895

[b66] Conant G. C. & Wolfe K. H. Turning a hobby into a job: how duplicated genes find new functions. Nat Rev Genet 9, 938–950 (2008). 19015656 10.1038/nrg2482

[b67] Conrad B. & Antonarakis S. E. Gene duplication: a drive for phenotypic diversity and cause of human disease. Annu Rev Genom Hum G 8, 17–35 (2007). 10.1146/annurev.genom.8.021307.110233 17386002

[b68] Bardwell J. C. & Craig E. A. Major heat shock gene of Drosophila and the Escherichia coli heat-inducible dnaK gene are homologous. Proc Natl Acad Sci U S A 81, 848–852 (1984). 6322174 10.1073/pnas.81.3.848 PMC344935

[b69] Lombard J., Lopez-Garcia P. & Moreira D. The early evolution of lipid membranes and the three domains of life. Nat Rev Microbiol 10, 507–515 (2012). 22683881 10.1038/nrmicro2815

[b70] Michell R. H. Inositol derivatives: evolution and functions. Nat Rev Mol Cell Biol 9, 151–161 (2008). 18216771 10.1038/nrm2334

[b71] Johnson M. *et al.* NCBI BLAST: a better web interface. Nucleic Acids Res 36, W5–9 (2008). 18440982 10.1093/nar/gkn201 PMC2447716

[b72] Katoh K. & Standley D. M. MAFFT multiple sequence alignment software version 7: improvements in performance and usability. Mol Biol Evol 30, 772–780 (2013). 23329690 10.1093/molbev/mst010 PMC3603318

[b73] Tamura K. *et al.* MEGA5: molecular evolutionary genetics analysis using maximum likelihood, evolutionary distance, and maximum parsimony methods. Molecular Biol Evol 28, 2731–2739 (2011). 10.1093/molbev/msr121 PMC3203626 21546353

[b74] Holm L. & Park J. DaliLite workbench for protein structure comparison. Bioinformatics (Oxford, England) 16, 566–567 (2000). 10.1093/bioinformatics/16.6.566 10980157

[b75] Jo S., Vargyas M., Vasko-Szedlar J., Roux B. & Im W. PBEQ-Solver for online visualization of electrostatic potential of biomolecules. Nucleic Acids Res 36, W270–275 (2008). 18508808 10.1093/nar/gkn314 PMC2447802

[b76] Cyr D. M. Cooperation of the molecular chaperone Ydj1 with specific Hsp70 homologs to suppress protein aggregation. FEBS Lett 359, 129–132 (1995). 7867784 10.1016/0014-5793(95)00024-4

[b77] Freeman B. C. & Morimoto R. I. The human cytosolic molecular chaperones hsp90, hsp70 (hsc70) and hdj-1 have distinct roles in recognition of a non-native protein and protein refolding. EMBO J 15, 2969–2979 (1996). 8670798 PMC450238

[b78] Dowler S., Kular G. & Alessi D. R. Protein lipid overlay assay. Sci STKE 2002, pl6 (2002). 11972359 10.1126/stke.2002.129.pl6

