# Supplementary material for: Functional Diversification and Specialization of Cytosolic 70-kDa Heat Shock Proteins
Source: Sci Rep. 2015 Mar 20;5:9363. doi: 10.1038/srep09363 (PMC4366816; doi:10.1038/srep09363)
Supplement: Supplementary Information — Supplementary Data [file srep09363-s1.pdf]

Functional Diversification and Specialization of Cytosolic 70-kDa Heat Shock Proteins

Chelsea McCallister[1], Matthew C. Siracusa[1], Farzaneh Shirazi[1], Dimitra Chalkia[2], and Nikolas Nikolaidis[1*]

[1] Department of Biological Science, Center for Applied Biotechnology Studies, and Center for Computational and Applied Mathematics, California State University, Fullerton, Fullerton, CA 92834

[2]Center for Mitochondrial and Epigenomic Medicine, The Children's Hospital of Philadelphia Research Institute, Philadelphia, PA

***Corresponding author:** Nikolas Nikolaidis, Department of Biological Science, Center for Applied Biotechnology Studies, and Center for Applied Mathematics, College of Natural Sciences and Mathematics, California State University Fullerton, Fullerton, CA 92834-6850; Tel: 657-278-4526; e-mail: nnikolaidis@fullerton.edu

Supplementary Material Online

Supplementary Tables S1-S2

Supplementary Figs. S1-S2

Supplementary Table S1. Binding kinetics of HspA1A (A1A) and HspA8 (A8) to PtdOH, PtdGro, GalCer-I^3-sulfate, Ptd2Gro

	PtdOH		PtdGro		GalCer-I^3-sulfate		Ptd2Gro	
	A1A	A8	A1A	A8	A1A	A8	A1A	A8
K_d	151±11.7	341±27	203±27.6	40±12.9	111±9.7	85±12.5	159±9.4	128±16.8
B_{max}	54.1±3.1	81.9±20.9	56.7±1.9	52±1.3	56.7±1.1	73.6±1.5	58.7±1.5	49.6±1.9
a	2.1±0.3	0.6±0.3	NA	NA	NA	NA	3.7±0.7	1.5±0.3
B_0	4.5±2.4	1.4±2.1	NA	NA	NA	NA	5.1±1.4	5.1±1.3

K_d (apparent dissociation constant), μM; B_{max} (maximal binding), % protein bound; a (Hill coefficient); B_0 (initial binding)

Supplementary Table S2. Binding models, equations, and regression values that best describe the interaction between HspA1A and HspA8 to different lipids

**PtdSer**			
	model	equation	R^2
**HspA1A**	Two sites saturation	$y = B_{max1} \times x/(K_{d1}+x)+B_{max2} \times x/(K_{d2}+x)$	0.9936
**HspA8**	One site saturation + Non specific	$y = B_{max} \times x/(K_d+x)+N_s \times x$	0.9906
**BMP**			
	model	equation	R^2
**HspA1A**	Hills 4 parameter	$y = B_0 + B_{max} \times x^a/(K_d^a+x^a)$	0.9915
**HspA8**	Hills 4 parameter	$y = B_0 + B_{max} \times x^a/(K_d^a+x^a)$	0.9921
**PtdOH**			
	model	equation	R^2
**HspA1A**	Hills 4 parameter	$y = B_0 + B_{max} \times x^a/(K_d^a+x^a)$	0.9534
**HspA8**	Hills 4 parameter	$y = B_0 + B_{max} \times x^a/(K_d^a+x^a)$	0.9763
**PtdGro**			
	model	equation	R^2
**HspA1A**	One site saturation	$y = B_{max} \times x/(K_d+x)$	0.9443
**HspA8**	One site saturation	$y = B_{max} \times x/(K_d+x)$	0.9616
**GalCer-I^3-sulfate**			
	model	equation	R^2
**HspA1A**	Hills 4 parameter	$y = B_0+B_{max} \times x^a/(K_d^a+x^a)$	0.9879
**HspA8**	One site saturation	$y = B_{max} \times x/(K_d+x)$	0.9801
**Ptd2Gro**			

	model	equation	R^2
**HspA1A**	Hills 4 parameter	$y = B_0+B_{max}\times x^a/(K_d^a+x^a)$	0.9900
**HspA8**	Hills 4 parameter	$y = B_0+B_{max}\times x^a/(K_d^a+x^a)$	0.9861

Supplementary figure legends

Supplementary Fig. S1. HspA1A and HspA8 have different surface charge. The figure depicts the distribution of the electrostatic potential on solvent-accessible surfaces of the HspA1A (left panel) and HspA8 (right panel) NBD structure. The electrostatic potential was calculated by solving the Poisson-Boltzmann equation using the PBEQ Solver tool. The regions colored in blue are associated with positively charged groups, red with negative charges, and white-gray areas are electrically neutral. Structures are shown in equivalent orientations and are presented as semi-transparent surfaces to visualize the secondary elements. Figures were generated with PyMol. The PDB codes for the structures used to generate the models are: 3JXU for HspA1A and 3HSC for HspA8.

Supplementary Fig. S2. Recombinant Hsp70s were pelleted in the presence of various concentrations of liposomes. Representative SDS-PAGE gel electrophoresis of the supernatant (S) and pellet (P) fractions of the vesicle sedimentation assays for both HspA1A and HspA8 using different concentrations (numbers in mM on top) for two different lipid mixtures [PtdCho and PtdCho:PtdSer (80:20 mol/mol)] at the right side of the gels).

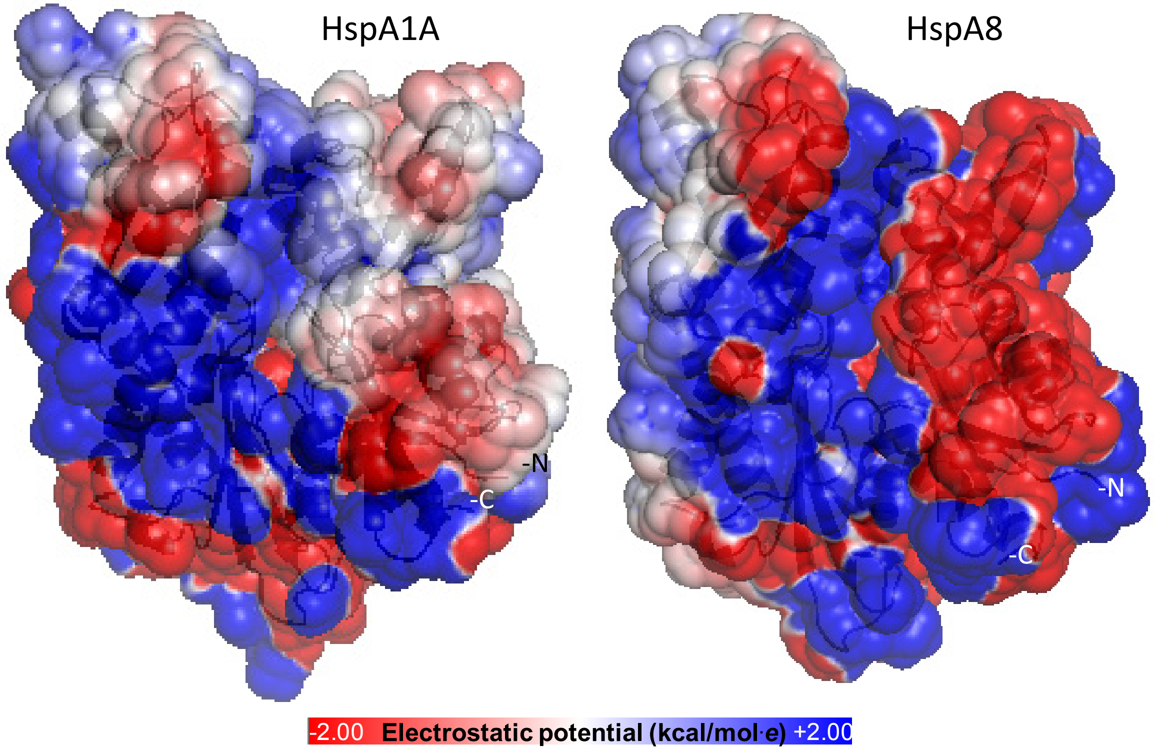

Supplementary Fig. S1

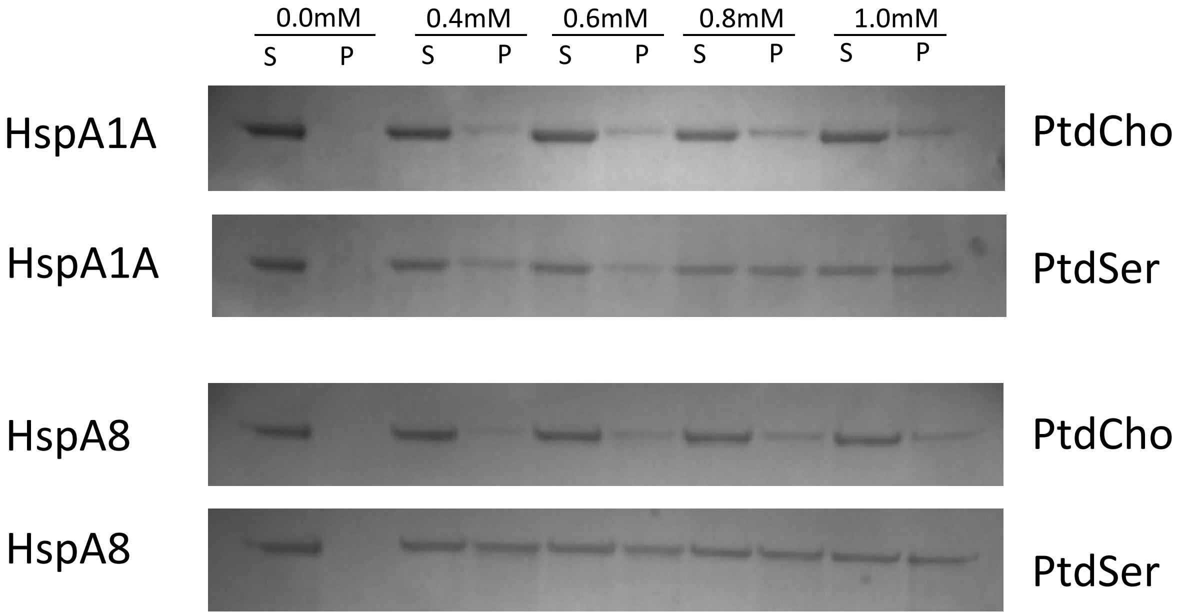

Supplementary Fig. S2
